# Out of the Dark and Into the Light: A New View of Phytochrome Photobodies

**DOI:** 10.3389/fpls.2021.732947

**Published:** 2021-08-31

**Authors:** Sarah A. Pardi, Dmitri A. Nusinow

**Affiliations:** ^1^Donald Danforth Plant Science Center, St. Louis, MO, United States; ^2^Division of Biology and Biomedical Sciences, Washington University in St. Louis, St. Louis, MO, United States

**Keywords:** phytochrome, photobodies, biomolecular condensates, liquid–liquid phase separation, intrinsically disordered protein

## Abstract

Light is a critical environmental stimulus for plants, serving as an energy source via photosynthesis and a signal for developmental programming. Plants perceive light through various light-responsive proteins, termed photoreceptors. Phytochromes are red-light photoreceptors that are highly conserved across kingdoms. In the model plant *Arabidopsis thaliana*, phytochrome B serves as a light and thermal sensor, mediating physiological processes such as seedling germination and establishment, hypocotyl growth, chlorophyll biogenesis, and flowering. In response to red light, phytochromes convert to a biologically active form, translocating from the cytoplasm into the nucleus and further compartmentalizes into subnuclear compartments termed photobodies. PhyB photobodies regulate phytochrome-mediated signaling and physiological outputs. However, photobody function, composition, and biogenesis remain undefined since their discovery. Based on photobody cellular dynamics and the properties of internal components, photobodies have been suggested to undergo liquid-liquid phase separation, a process by which some membraneless compartments form. Here, we explore photobodies as environmental sensors, examine the role of their protein constituents, and outline the biophysical perspective that photobodies may be undergoing liquid-liquid phase separation. Understanding the molecular, cellular, and biophysical processes that shape how plants perceive light will help in engineering improved sunlight capture and fitness of important crops.

## Introduction

Light is the most critical environmental stimulus for all plant development, serving as the energy source for photosynthesis and as an environmental cue to regulate growth and development. Thus, it is critical for plants to appropriately detect, coordinate, and respond to light cues for their overall fitness and survival. To perceive light, plants have evolved different classes of photoreceptors that absorb light wavelengths from the UV to far-red (380–735 nm wavelengths), including UV RESISTANCE LOCUS 8 (UVR8), PHOTOTROPINS, CRYPTOCHROMES, LOV (Light, Oxygen, Voltage)-KELCH DOMAIN containing F-box proteins, and PHYTOCHROMES (Butler et al., 1959; Gressel, 1979; Kandori et al., 1992; Jansen et al., 1998; Briggs and Huala, 1999; Cashmore et al., 1999; Lin, 2000; Nelson et al., 2000; Somers et al., 2000; Briggs et al., 2001; Jarillo et al., 2001; Kinoshita et al., 2001; Kagawa et al., 2001; Sakai et al., 2001; Schultz et al., 2001; Briggs and Christie, 2002; Kasahara et al., 2002; Lin, 2002; Frohnmeyer and Staiger, 2003; Imaizumi et al., 2003; Kleine et al., 2003; Ulm and Nagy, 2005; Kaiserli and Jenkins, 2007; Quail, 2010; Yu et al., 2010; Chaves et al., 2011; Rizzini et al., 2011; Jenkins and Brown, 2018).

Phytochromes perceive red/far-red light (600–750 nm) and regulate many aspects of plant development, including seed germination, de-etiolation, gravitropism, flowering, circadian rhythms, and senescence (Bae and Choi, 2008; Franklin and Quail, 2010; Kami et al., 2010; Paik and Huq, 2019). In the model plant *Arabidopsis thaliana*, phytochromes are a five-member family, phyA-phyE (Jones and Quail, 1989; Somers et al., 1991; Quail, 1991; López-Juez et al., 1992; Clack et al., 1994; Mathews, 2010). PhyA is classified as light-labile and is the most abundant phytochrome in etiolated seedlings, whereas phyB-E are classified as light-stable (Clack et al., 1994; Nagy and Schäfer, 2002). PhyA is mainly responsible for sensing and responding to far-red light, in addition to red light, whereas phyB-E are responsible for photomorphogenesis in response to red light and foliar shade (Whitelam et al., 1992; McCormac et al., 1993; Nagatani et al., 1993; Parks and Quail, 1993; Whitelam et al., 1993; Reed et al., 1994; Paik and Huq, 2019). Phytochrome A and B have overlapping and distinct photosensory roles in seedling development (Reed et al., 1994).

Phytochromes are dimeric chromoproteins, with each monomer covalently attached to a light-absorbing linear tetrapyrrole chromophore, phytochromobilin (Cornejo et al., 1992; Terry et al., 1995). The protein domains in plant phytochromes can be divided into two modules: the chromophore-bearing, N-terminal photosensory module, responsible for light perception and signaling, and the C-terminal module that directs nuclear localization, dimerization, and nuclear body formation (Rockwell et al., 2006; Nagatani, 2010). Phytochromes exist in two stable conformers: a biologically inactive red-light absorbing form (Pr) and a biologically active far-red light-absorbing form (Pfr) (Rockwell et al., 2006; Bae and Choi, 2008). The phytochrome holoprotein is assembled in the cytosol in the inactive Pr conformation. Once converted to Pfr in response to red light, phytochromes move from the cytoplasm into the nucleus, where most signaling functions occur (Sakamoto and Nagatani, 1996; Kircher et al., 1999, 2002; Yamaguchi et al., 1999; Kim et al., 2000).

Phytochrome function is dependent on its localization in the nucleus (Huq et al., 2003; Matsushita et al., 2003; Genoud et al., 2008). Phytochrome A-E have differing mechanisms by which they are transported to the nucleus. Of the phytochromes, phyA nuclear localization is well characterized and is dependent on FAR-RED ELONGATED HYPOCOTYL 1 (FHY1) and FHY1-LIKE (FHL) (Hiltbrunner et al., 2006; Genoud et al., 2008; Helizon et al., 2018). FHY1 and FHL act as shuttle proteins, binding to phyA-Pfr in the cytoplasm and transporting it to the nucleus (Hiltbrunner et al., 2006; Genoud et al., 2008; Rausenberger et al., 2010; Helizon et al., 2018). An NLS-like motif in the C-terminal domain of phyB-E is sufficient for localizing these phytochromes to the nucleus (Sakamoto and Nagatani, 1996; Chen et al., 2005), and the C-terminal module is necessary for nuclear localization (Matsushita et al., 2003; Chen et al., 2005). PHYTOCHROME INTERACTING FACTOR 3 (PIF3) (Pfeiffer et al., 2012) and SUPPRESSOR OF PHYA-105 (SPA1) (Zheng et al., 2013) have been shown to promote nuclear import of phyB-Pfr. However, the transport mechanism remains to be defined experimentally. PhyB nuclear localization may involve complementary binding partners under varying light conditions. Nuclear localization of phyC, phyD, and phyE remain the least understood (Ádám et al., 2013; Klose et al., 2015b). Future studies are required to define which proteins, if any, are not only sufficient but necessary for transporting phyB-E.

Phytochrome signaling is responsive to temperature in addition to light, since the Pfr form is thermally unstable. Phytochromes undergo isomerization from the active Pfr form to the inactive Pr state in a light-independent, temperature-dependent process called dark or thermal reversion (Jung et al., 2016; Legris et al., 2016). (For a review on phytochrome thermal reversion, readers are pointed to Klose et al., 2020). Two studies showed that phytochromes serve as temperature sensors through their thermal reversion capability. Using genomic approaches, Jung et al. demonstrated that phytochromes alter the *Arabidopsis* transcriptome in response to warm temperatures (Jung et al., 2016). Specifically, temperature affects phyB’s ability to bind to target genes’ promoters and repress PIF4 activity. PIFs are antagonists of phytochromes, promoting hypocotyl growth (Ni et al., 1998; Oh et al., 2007; Shen et al., 2008; Lorrain et al., 2009; Shin et al., 2009; Leivar and Quail, 2011). Additionally, they showed that phyB’s dark reversion integrates temperature signals during the night (Jung et al., 2016). Legris et al. (2016) demonstrated in a complementary study – through genetics, biochemical measurement of phyB Pfr:Pr isomerization, and modeling approaches – that increased temperature reduces the amount of phyB Pfr pool and strength of signaling. In addition, a negative correlation was shown between temperature and phyB activity. Overall, these two breakthrough studies concluded that in addition to functioning as a light sensor, phyB is a thermosensor in plants (Jung et al., 2016; Legris et al., 2016), providing critical mechanistic insight into how plants perceive warm temperatures. This insight shapes future research on light signaling to mitigate the harmful effects of increasing global temperature on agriculturally important crops.

A critical step in phytochrome signaling is the assembly of active phytochrome Pfr into large (>500 nm) subnuclear membraneless compartments termed photobodies (Yamaguchi et al., 1999; Chen et al., 2003; Huang et al., 2016). Increasing the intensity of red light, which stabilizes the Pfr form, promotes the formation of large photobodies (Chen et al., 2003; Van Buskirk et al., 2012). Conversely, conditions that revert Pfr to Pr, such as far-red light, high temperature, or darkness, cause photobodies to disassemble into many smaller foci and ultimately dissipate within the nucleoplasm (Kircher et al., 1999; Yamaguchi et al., 1999; Chen and Chory, 2011; Van Buskirk et al., 2012, 2014). Photobody localization is conserved in dicots and monocots (Kircher et al., 1999, 2002; Kim et al., 2000). Recent work supports that photobodies are an essential cellular structure for phyB signaling (Su and Lagarias, 2007; Chen et al., 2010; Kaiserli et al., 2015; Huang et al., 2016, 2019; Enderle et al., 2017; Qiu et al., 2017; Yang et al., 2019; Yoo C.Y. et al., 2019). There are several hypothesized functions of photobodies, and multiple proteins have been shown to colocalize with photobodies (Holm et al., 2002; Bauer et al., 2004; Subramanian et al., 2004; Al-Sady et al., 2006; Hiltbrunner et al., 2006; Jang et al., 2007; Yu et al., 2008; Liu et al., 2011; Yan et al., 2011; Zuo et al., 2011; Van Buskirk et al., 2012). However, photobody function, protein components, and biogenesis are yet to be clearly defined.

Below, we explore the biological significance of photobodies in *Arabidopsis thaliana*, how photobodies contribute to signaling in fluctuating environments, protein components that promote photobody formation, photobody biogenesis, how liquid–liquid phase separation (LLPS) may underlie the biophysical mechanism of assembly, and photobody functions. Lastly, we discuss some of the many exciting directions for future research on LLPS of photobodies.

## Discovery and Biological Significance

Subnuclear structures of phytochrome were first observed in 1999 in transgenic plants overexpressing phyB-GFP (Yamaguchi et al., 1999). Different nomenclature has been used throughout the years to describe these subnuclear compartments: speckles, foci, nuclear bodies, and ultimately photobodies. Using immunoblotting and various microscopy tools, Yamaguchi et al. reported the light-dependent subcellular distribution of phyB-GFP. This fusion protein localized to the nucleus and further compartmentalized into foci with a size of ∼1 μm. They also observed a positive correlation between the duration of red-light exposure and the size of the phyB-GFP photobody. The authors compared these phyB nuclear bodies to promyelocytic leukemia (PML) bodies, which are involved in RNA metabolism and transcription regulation (Stuurman et al., 1990; Lallemand-Breitenbach, 2010). This was the first description connecting phyB photobodies to nuclear condensates found in other systems. Since this fundamental study used a highly active Cauliflower Mosaic Virus 35S promoter to express phyB-GFP, it was suggested that the existence of these structures was merely due to overexpression (Yamaguchi et al., 1999). However, electron microscopy experiments using immuno-gold labeling of phyA in *Arabidopsis* demonstrated that endogenous phytochromes formed photobodies (Sheerin et al., 2015). Further work found that all members of the phy family formed nuclear bodies at differing rates in response to red and white light (Kircher et al., 2002), suggesting that photobody localization was a regulated process. Thus, the subnuclear assembly of phyB-GFP is presumed to reflect endogenous phyB localization (Yamaguchi et al., 1999; Gil et al., 2000; Kim et al., 2000; Kircher et al., 2002).

Even though these studies demonstrated that photobodies are not artifacts of exogenous expression, their biological importance remained in question. Matsushita et al. overexpressed phyB’s N-terminal domain fused to a nuclear localization sequence (NLS) and a dimerization domain (Matsushita et al., 2003). They found that this phyB chimera did not form photobodies but was sufficient for phyB signaling, rescuing several phyB physiological responses in constant red-light conditions (Matsushita et al., 2003). Thus, the authors concluded that nuclear localization, not photobody formation, was necessary for phyB signaling (Matsushita et al., 2003). Furthermore, under low red-light conditions where phyB is dispersed throughout the nucleoplasm rather than localized to photobodies, plants show physiological responses reflecting the presence of active phyB. Thus, nucleoplasmic phyB is sufficient for signaling in these conditions (Parks and Quail, 1993; Wagner et al., 1996; Gil et al., 2000; Krall and Reed, 2000; Chen et al., 2003). It is possible that photobodies may promote certain phyB functions but not others, or that photobodies may act to enhance phyB activity through its N-terminal module. In agreement with this, Chen et al. (2003) proposed a model in which phyB is active both when dispersed throughout the nucleoplasm and when localized to photobodies, with different phyB mechanisms responding to varying intensities of red light.

Although the studies above argue that photobodies are dispensable, accumulating evidence supports the idea that photobodies are essential for phyB signaling, as discussed below. *PhyB-GFP* mutants that are nuclear-localized but do not form nuclear bodies have impaired light signaling, demonstrating a strong correlation between phyB biological activity and compartmentalization into photobodies (Su and Lagarias, 2007; Chen et al., 2010; Kaiserli et al., 2015; Huang et al., 2016, 2019; Enderle et al., 2017; Qiu et al., 2017; Yang et al., 2019; Yoo C.Y. et al., 2019). Although Matsushita et al. (2003) showed that photobodies are dispensable in constant red light, a study by Van Buskirk et al. (2014) demonstrated the role of photobodies under more natural light/dark conditions. Using *PhyB* mutants, a strong link was shown between photobodies and phyB regulated processes after dusk. Specifically, the presence of photobodies was highly correlated with hypocotyl elongation inhibition in the dark and PIF3 degradation (Van Buskirk et al., 2014). During the day, phytochromes promote photomorphogenesis and inhibit hypocotyl elongation by repressing PIF activity via proteasome-mediated degradation (Al-Sady et al., 2006; Leivar et al., 2009; Shin et al., 2009; Leivar and Quail, 2011). When photobodies disassemble, PIF3 is no longer repressed, and hypocotyl growth is promoted (Van Buskirk et al., 2014). In agreement, Qiu et al. (2017) demonstrated that expression of phyB’s C-terminal module in stable transgenic lines forms photobodies and is sufficient for degrading PIF3 and regulating a subset of PIF-dependent genes. Challenging a previous model in which phyB’s N-terminal module was responsible for PIF3’s light-induced degradation, they demonstrated that PIF3’s degradation is dependent on its interaction with the dimeric C-terminal module (Shimizu-Sato et al., 2002; Oka et al., 2008; Kikis et al., 2009; Leivar and Quail, 2011; Qiu et al., 2017). Additionally, the significance of photobodies can be seen at the phenotypic level, with phyB photobody localization patterns being tightly correlated with the degree of hypocotyl elongation. Pfr, which promotes the formation of large photobodies, produces a short hypocotyl phenotype, whereas when photobodies are small or dispersed in low red:far-red (R:FR) light, seedlings are taller (Chen et al., 2003). Overall, there is growing acceptance that photobodies are essential for phyB responses (Kircher et al., 2002; Chen et al., 2003; Oka et al., 2008).

## Photobodies Are Important for Environmental Sensing and Signaling

**FIGURE 1:**
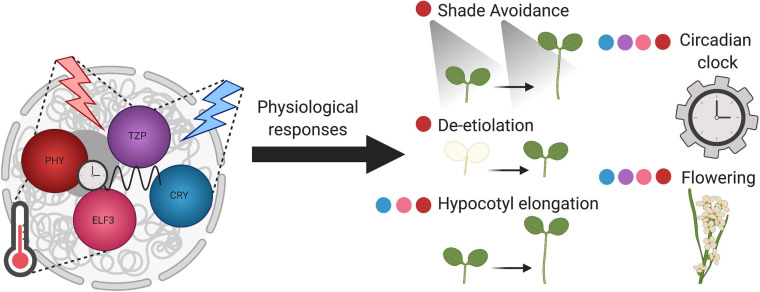
Plant nuclear bodies coordinate environmental input with physiological responses. Phytochrome B photobodies (red), TZP (purple), CRY (blue), and ELF3 (pink) bodies, perceive different environmental stimuli. Phytochrome photobodies and TZP both respond to red light, whereas CRY bodies respond to blue light. TZP integrates red and blue light signaling. PhyB photobodies and ELF3 bodies are sensitive to temperature cues. Phy, TZP, CRY, and ELF3 bodies sense circadian clock input signals. The components of these nuclear bodies transduce environmental signals to diverse physiological outputs, represented by the colored dots adjacent to the developmental process. Overall, these plant nuclear bodies act as environmental sensors.

Photobody formation is responsive to the external environment, as is described below. They are found to be important for light, circadian, and temperature signaling, potentially acting as hubs connecting these signaling pathways and serving as environmental sensors in plants (Kircher et al., 2002; Chen et al., 2003; Huang et al., 2019; Hahm et al., 2020; Figure 1).

### Light Signaling

Photobody formation responds to specific wavelengths and intensities of light (Kircher et al., 1999; Yamaguchi et al., 1999; Kim et al., 2000; Kircher et al., 2002; Chen et al., 2003). The amount of phyB-Pfr to total phyB and the formation of photobodies depends on the intensity of red light and the ratio of R:FR. The size and distribution patterns of photobodies correlates with the intensity of red light. As red light intensity increases from 0.5 μmol⋅m^–2^⋅sec^–1^ to 8 μmol⋅m^–2^⋅sec^–1^, PhyB-GFP is first evenly dispersed within the nucleoplasm, then hundreds of small photobodies form, then large photobodies (∼1 μm) appear along with the small photobodies, and finally, large photobodies exclusively form within the nucleus. Thus, the increase in light intensity in a dosage dependent manner and the resulting photoconversion and nuclear localization of Pfr correlates with the appearance of large photobodies (Chen et al., 2003). Within minutes of initial light exposure, phyA and phyB rapidly assemble into many small early ‘transient’ bodies, but after extended light exposure, a few large ‘stable’ bodies form, mainly made up of phyB (Casal et al., 2002; Kircher et al., 2002). These larger, stable bodies are referred to as ‘photobodies,’ which are correlated with functional phyA and phyB activity (Kircher et al., 1999, 2002; Yamaguchi et al., 1999; Kim et al., 2000). Under red light, PIF3 transiently colocalizes with these early phyB photobodies, where it then gets degraded in a light-dependent manner (Bauer et al., 2004). In the biogenesis of photobodies, it is likely that early transient bodies merge into the larger stable photobodies. Overall, the amount of phyB-Pfr to total phyB, controlled by red light, has to surpass a critical threshold to form photobodies (Chen et al., 2003).

Changing R:FR ratios, responsible for shade avoidance responses, has the same effect on photobody formation as light intensity (Casal et al., 2002; Chen et al., 2003; Trupkin et al., 2014). Low R:FR reduces phyB-Pfr to total phyB, which reduces the number of large photobodies and leads to the formation of many small photobodies (Trupkin et al., 2014). This pattern is reversible—when plants are transferred back from either low to high irradiance or R:FR, large photobodies form, demonstrating their dynamic behavior (Trupkin et al., 2014). Overall, these nuclear bodies may potentially act as light sensors, forming and dissipating in response to specific wavelengths and intensities of light.

### Circadian Regulation

The circadian clock may regulate photobody dynamics. In agreement with being light-responsive, photobody accumulation increases over the day (Kircher et al., 2002). Surprisingly, when grown under short-day conditions (8 h light:16 h dark), phyA, phyB, phyC, and phyE-GFP nuclear body number increased significantly before dawn in anticipation of the subjective light period (Kircher et al., 2002). Based on these findings, it was concluded that photobodies are modulated by the circadian clock (Kircher et al., 2002). However, it remains unclear what mechanism allows for the circadian-regulated dispersal and reassembly of phy-Pfr photobodies.

In addition to being regulated by the clock, phytochromes also signal into the plant circadian clock (Somers et al., 1998; Martínez-García et al., 2000; Hu et al., 2013). A recent study demonstrated that photobodies are important for the entrainment of the circadian clock oscillator (Huang et al., 2019). This study utilized the phyB constitutively active mutant phyB^Y276H^ (YHB) to specifically activate phyB signaling while keeping other photoreceptors turned off in the dark (Hu et al., 2009). YHB is sufficient for maintaining circadian oscillations of a Luciferase reporter under darkness, a condition that typically leads to dampening of clock rhythms in wild-type plants (Jones, 2009). When YHB is present in a mutant background that prevents photobody formation, YHB’s constitutively photomorphogenic phenotype and light input into the circadian clock are abolished (Huang et al., 2019). Specifically, this line without photobodies could not sustain circadian rhythms in constant darkness, even though the YHB allele locked phyB in its active state (Huang et al., 2019). Together, these studies demonstrate that photobody dynamics are both an input and output of the plant circadian clock.

### Temperature Perception

Not only do photobodies sense light and circadian cues, photobodies also act as temperature sensors through phyB’s thermal reversion ability (Jung et al., 2016; Legris et al., 2016). Thermal reversion causes the pool of Pfr to be reduced in warmer temperatures and thus decreases the size of photobodies (Legris et al., 2016). In response to temperature, photobodies within a nucleus can vary in localization patterns, stabilities, and tissue-specific dynamics (Hahm et al., 2020). Individual photobodies have distinct thermostabilities; in response to warm temperatures, thermosensitive photobodies rapidly disassemble, while thermo-insensitive photobodies remain unaltered (Hahm et al., 2020). Hahm et al. (2020) also found that some photobodies are located adjacent to the nucleolus, termed nucleolar associated photobodies, while others were found distributed throughout the nucleus, termed non-nucleolar associated photobodies. The non-nucleolar associated photobodies were found to be thermosensitive, while the nucleolar associated photobodies were thermo-insensitive, suggesting a connection between thermostability and photobody position within the nucleus (Hahm et al., 2020). Another recent study provided evidence that increasing temperatures decreases the size of photobodies during the night, and that phyB can transfer night-time temperature information to influence the next day’s hypocotyl growth (Murcia et al., 2020). Lastly, genetic evidence demonstrated that the hypocotyl growth of *Arabidopsis* lacking photobodies was hypersensitive to high temperature under long days, suggesting photobodies affect thermoresponsiveness (Huang et al., 2019). These studies provide strong evidence that photobody assembly and disassembly are highly responsive to fluctuating temperatures. In sum, photobodies are important for sensing and responding to diverse environmental cues, particularly light, circadian, and temperature signals (Figure 1).

## Protein Components Regulating Photobody Formation

There are multiple protein components thought to make up photobodies, many of which are involved in light signaling through gene regulation or proteolysis (Holm et al., 2002; Bauer et al., 2004; Subramanian et al., 2004; Al-Sady et al., 2006; Hiltbrunner et al., 2006; Jang et al., 2007; Yu et al., 2008; Liu et al., 2011; Yan et al., 2011; Zuo et al., 2011; Van Buskirk et al., 2012). However, the term ‘photobody’ does not necessarily encompass all the various potential components into a single entity. There are likely distinct nuclear bodies forming in response to different environmental stimuli. While many proteins colocalize to photobodies, thus far, there are only a few that are shown to regulate phyB-photobody formation.

HEMERA (HMR), also known as pTAC12, is involved in proteolysis and transcription and was the first protein component to be identified as essential for photobody formation (Chen et al., 2010; Nevarez et al., 2017). The *hmr* mutant has an albino and long hypocotyl phenotype, indicating its involvement in chloroplast biogenesis and red light signaling. Ultimately, HMR couples nuclear and chloroplastic gene expression (Chen et al., 2010). HMR localizes in chloroplasts, functioning as an essential plastid-encoded plastid RNA polymerase (PEP) component and as an activator of PEP-mediated plastid-encoded photosynthetic genes. HMR then relocates into the nucleus where it modulates phy-PIF signaling (Pfalz et al., 2006; Chen et al., 2010; Steiner et al., 2011; Nevarez et al., 2017). The *hmr* mutant displays phyB photobodies that are either smaller in size or phyB-GFP dispersed throughout the nucleoplasm, demonstrating that HMR promotes phyB photobody formation (Chen et al., 2010). HMR is not only essential for photobody formation but is also required for phyA, PIF1, and PIF3 degradation (Chen et al., 2010; Galvão et al., 2012). The binding of phyB Pfr with HMR promotes the accumulation of HMR protein, which is required for PIF3 degradation in the dark (Galvão et al., 2012). In addition to mediating PIF1 and PIF3 protein degradation, HMR alters PIFs regulation of target genes (Galvão et al., 2012). Specifically, HMR directly interacts with PIF1 and PIF3 as a transcriptional coactivator to regulate a class of PIF target genes (Qiu et al., 2015). These studies propose that photobodies are sites for proteasomal degradation and provide evidence that supports the tight correlation between photobody formation and phyB function (Chen et al., 2010).

The second protein identified as essential for phyB photobody formation was PHOTOPERIODIC CONTROL OF HYPOCOTYL 1 (PCH1). PCH1 and its homolog PCH1-LIKE (PCHL) directly bind to phyB, phyD, and phyE, preferentially interacting with the Pfr form (Huang et al., 2016; Enderle et al., 2017). Under short-day conditions, *pch1 Arabidopsis* seedlings have an elongated hypocotyl phenotype, similar to *phyB*’s elongated hypocotyl phenotype (Huang et al., 2016). PCH1 and PCHL inhibit and slow phyB thermal reversion *in vivo* and *in vitro*, stabilizing phyB-Pfr (Enderle et al., 2017; Huang et al., 2019). The constitutively active phyB mutant, YHB, showed that PCH1 is essential for phyB photobody formation and serves as a structural component *in vivo* (Huang et al., 2019). While the overexpression of PCHL increases photobody formation, PCHL is not required for photobody formation. In the *pch1* phyB-GFP mutant, either one photobody is formed or phyB-GFP signal is dispersed within the nucleus. Both HMR and PCH1 are involved in temperature sensing, with HMR being required for phyB daytime temperature sensing through PIF4 (Huang et al., 2019; Qiu et al., 2019). Affinity Purification Mass Spectrometry (AP-MS) data of PCH1 protein interactions shows an overlap of PCH1 interacting partners with other photobody components, such as phyA-E, CONSTITUTIVE PHOTOMORPHOGENIC 1 (COP1), SPA1, EARLY FLOWERING 3 (ELF3), and TANDEM ZINC-FINGER PLUS3 (TZP), suggesting PCH1 and PCH1-interacting proteins are bona fide photobody components (Huang et al., 2016). Interestingly, PCH1 co-immunoprecipitation data did not identify HMR as an interacting partner, suggesting PCH1-mediated phyB photobodies may be distinct from HMR-mediated phyB photobodies (Huang et al., 2016).

In two recent studies, the paralogs REGULATOR OF CHLOROPLAST BIOGENESIS (RCB) and NUCLEAR CONTROL OF PEP ACTIVITY (NCP) were identified in a forward genetic screen as plastid and nuclear-localized proteins, similar to HMR. Importantly, they were shown to regulate phyB photobody biogenesis (Yang et al., 2019; Yoo C.Y. et al., 2019). Similar to HMR, RCB, and NCP first localize to plastids, then translocate to the nucleus where they participate in phyB signaling. Furthermore, RCB and NCP also promote the formation of large photobodies and the degradation of PIF1 and PIF3 in the nucleus (Yoo C.Y. et al., 2019). Similar to *hmr* phyB-GFP, the *rcb* phyB-GFP and the *ncp* phyB-GFP mutants cannot assemble into large photobodies, but instead form many smaller nuclear bodies (Yang et al., 2019; Yoo C.Y. et al., 2019). RCB is involved in the same phyB pathway as HMR, binds to Pfr and Pr of phyA and phyB, and modulates chloroplast biogenesis via PIF degradation (Yoo C.Y. et al., 2019). While NCP plays an essential role in chloroplast biogenesis by regulating PEP assembly, RCB is not required for chloroplast biogenesis (Yang et al., 2019). These two studies proposed a model which connects phyB photobody biogenesis in the nucleus to plastid anterograde signaling, ultimately connecting photomorphogenesis with de-etiolation (Yang et al., 2019; Yoo C.Y. et al., 2019). The functions of photobody components support the importance of photobodies in phy-PIF mediated physiological responses.

In addition to phytochromes forming nuclear bodies, some phytochrome interacting proteins are sufficient for forming nuclear bodies. One example is TZP, which forms nuclear bodies in a red light and phyB-dependent manner (Kaiserli et al., 2015; Figure 1). These TZP-phyB bodies are dynamic, diurnally regulated, and associate with RNA (Kaiserli et al., 2015). TZP’s association with RNA infers that these bodies may be involved in RNA metabolism, a function of many nuclear bodies that contain RNA binding proteins (reviewed in Sabari et al., 2020). Interestingly, TZP-phyB bodies do not colocalize with HMR (Kaiserli et al., 2015). Thus, similar to HMR-phyB photobodies being distinct from PCH1-phyB photobodies, HMR-phyB photobodies may be separate from TZP-phyB photobodies.

Circadian clock proteins, such as ELF3 and GIGANTEA (GI), and proteins involved in light signaling, such as CRYPTOCHROME 1 (CRY1), CRY2, and E3 ubiquitin ligase COP1, also form nuclear bodies (Ang et al., 1998; Wang et al., 2001; Yu et al., 2009; Kim et al., 2013; Figure 1). ELF3, a member of the *Arabidopsis* EVENING COMPLEX and interactor of phyB, transmits light and temperature signals to the circadian clock, potentially through its ability to form nuclear bodies (Jung et al., 2020). ELF3 nuclear bodies colocalize with TZP, suggesting their connection in circadian signaling (Kaiserli et al., 2015). CRY1 and CRY2 form nuclear bodies in response to blue light intensity (Yu et al., 2009; Gu et al., 2012; Figure 1). For further reading on other examples of plant nuclear bodies, readers are pointed to the following excellent reviews (Ronald and Davis, 2019; Emenecker et al., 2020; Meyer, 2020). Based on their protein components, phyB photobodies, and other nuclear bodies, may have both shared and distinct functions.

## Photobody Biogenesis

Another long-standing question is the mechanism by which photobodies form. However, studies directly testing photobody biogenesis are limited. Thus far, synthetic biology and mathematical modeling have been the main approaches taken in elucidating their association.

The first study to assess how photobodies form utilized a nucleolus-tethering system (NoTS) (Liu et al., 2014). They designed their NoTS based on the bacterial LacO/LacI tethering system used previously to study nuclear body assembly in mammalian cells (Kaiser et al., 2008; Mao et al., 2011; Shevtsov and Dundr, 2011; Dundr, 2013). The NoTS artificially tethers a protein of interest to the nucleolus by fusion with a nucleolus marker protein (Liu et al., 2014). This allows the visualization of components localizing to and initiating nuclear body formation (Liu et al., 2014). They showed that these tethered photobodies are functional and found that the efficiency of phyB to initiate photobody formation was lower than that of other proteins, such as COP1, CRY1, and CRY2 (Liu et al., 2014). The lower efficiency of phyB to initiate assembly suggests that other factors assist phyB in photobody formation (Chen et al., 2010; Huang et al., 2019; Yang et al., 2019; Yoo C.Y. et al., 2019). Overall, this study concluded that since multiple photobody-localized proteins are sufficient to form nuclear bodies, photobodies form via self-organization (Liu et al., 2014).

**FIGURE 2:**
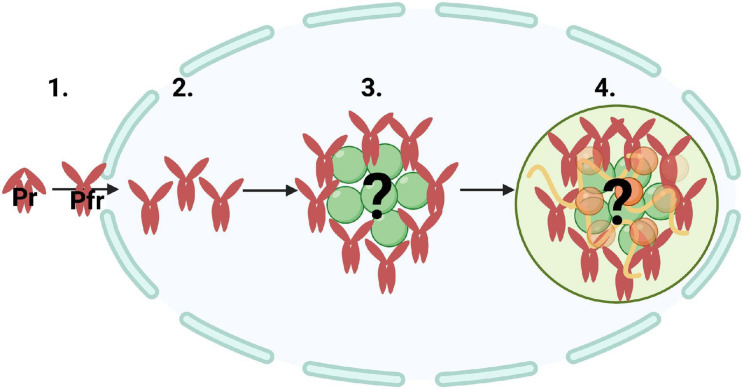
A potential mechanism of photobody biogenesis via nucleation and ultimately through liquid-liquid phase separation. Illustration of (1) inactive phytochrome (Pr) converting to active phytochrome (Pfr) and traveling into the nucleus, (2) nucleation of Pfr, (3) Pfr accumulating around an unknown core structural component or ‘seed,’ and (4) localizing into a single photobody. Phytochrome photobodies may be undergoing an initial nucleation step, which can initiate protein phase transitions, followed by more complex molecular interactions with other proteins (orange and green spheres) and nucleic acids (yellow curved lines).

Mathematical modeling has been another approach in understanding how photobodies form. Previous studies used mathematical modeling to assess the effect of phyB-Pfr conformation and dimerization on photobody association and dissolution (Rausenberger et al., 2010; Klose et al., 2015a). One study used mathematical modeling and statistical physics to understand the mechanism of phyB photobody biogenesis (Grima et al., 2018). Although their model assumed that photobodies are solely made up of phyB dimers, which is not the case *in vivo* (Van Buskirk et al., 2012), their calculations suggested that the kinetics of building photobodies could not simply reflect the assembly phyB dimers (Grima et al., 2018). Therefore, they reasoned that photobody formation consists of two steps: a fast nucleation step in which phyB aggregates or binds to other proteins, followed by a slower step of more complex binding. Grima et al. (2018) also suggested that photobodies may be hollow, which has since been supported by microscopy evidence (Perrella et al., 2021). In this case, phyB may bind to a structural component, also referred to as a seed component, to initiate formation (Mao et al., 2011; Shevtsov and Dundr, 2011; Figure 2). Since phytochromes homodimerize and heterodimerize with each other (Sharrock and Clack, 2004), phyB may first form small aggregates, followed by the slower process of more complex associations with other proteins and or nucleic acids (Figure 2). Although there are a few studies on photobody formation utilizing synthetic biology and mathematical modeling, another approach to study this cellular process may be through the lens of biophysics.

## Photobodies May Undergo Liquid–Liquid Phase Separation

Photobodies have been described as plant-specific biomolecular condensates (Cuevas-Velazquez and Dinneny, 2018; Emenecker et al., 2020). Biomolecular condensates are subcellular, membraneless compartments that concentrate biomolecules, such as proteins and nucleic acids, to organize cellular processes (Spector, 2006; Banani et al., 2017). Although terminology varies for these membraneless compartments, biomolecular condensate is a general term to describe the assembly of biomolecules, regardless of material properties or function (Banani et al., 2017). Many examples connect biomolecular condensates to neurodegenerative diseases, such as Alzheimer’s, amyotrophic lateral sclerosis (ALS), Huntington’s, and certain cancers (Spannl et al., 2019). Thus, understanding their biogenesis is of significance in the medical field. Studies on the assembly of biomolecular condensates in fungal and mammalian systems are extensive but are less developed in plant systems.

**FIGURE 3:**
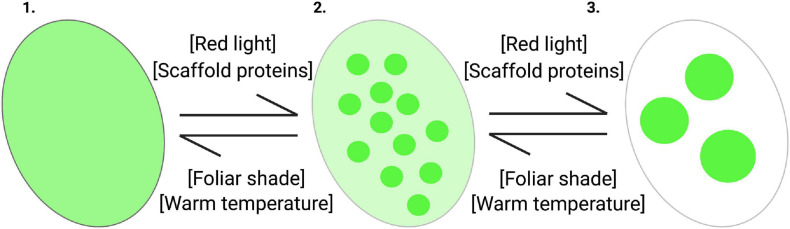
Phytochrome photobodies are a controllable system for studying factors that influence liquid-liquid phase separation in vivo. Within Arabidopsis nuclei (ovals), active phytochrome can transition from a single diffuse phase throughout the nucleoplasm (1) into two distinct phases: one with a low concentration that is evenly distributed throughout the system and a denser phase that takes the shape of liquid droplets, or photobodies (2), and finally into large photobodies (3). Green represents nuclear phy-Pfr, and the green circles represent phytochrome photobodies. Environmental and cellular factors influence the formation and dissipation of phytochrome photobodies. Increasing red-light intensity and the concentration of presumptive scaffold proteins, such as HMR, PCH1, RCB, and NCP, promote the formation of photobodies. Conversely, warm temperatures and low red:far-red ratios under foliar shade conditions promote the dissipation of photobodies.

A biophysical process by which condensates can form is through LLPS (Hyman et al., 2014). LLPS is the biophysical process by which two distinct liquid phases are formed, or demixed, into a dense phase and a less dense phase (Boeynaems et al., 2018; Posey et al., 2018; Gomes and Shorter, 2019; Figure 3). The key characteristics of condensates undergoing LLPS are that they display liquid-like properties: they are spherical, can fuse and relax like liquid droplets, and are dynamic – able to rapidly exchange with the surrounding cellular environment (Alberti et al., 2019). Previous fluorescence recovery after photobleaching (FRAP) experiments on phyB-YFP demonstrate that phyB photobodies are, in fact, dynamic (Rausenberger et al., 2010). Biomolecular condensates do not necessarily need to be liquid-like, they can be gel-like or take on a solid material state depending on the properties of the resident molecules.

**FIGURE 4:**
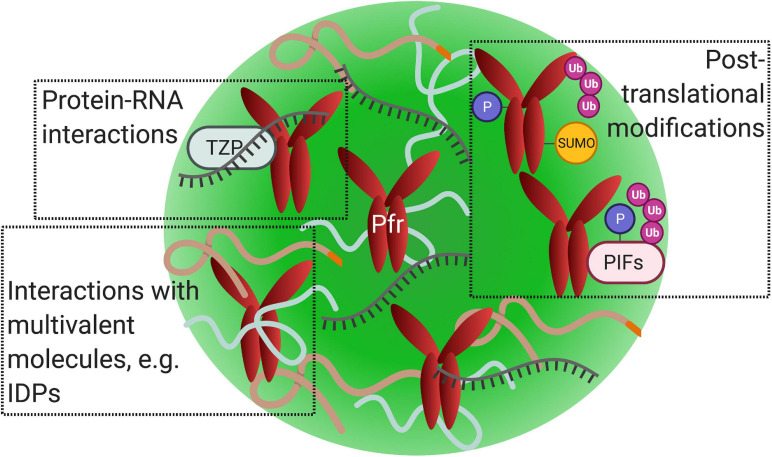
Phytochrome photobodies may share features associated with other nuclear biomolecular condensates. Illustration of active phytochrome (Pfr) within a single photobody (green sphere). Factors that promote nuclear condensate formation via liquid-liquid phase separation, including protein–protein interactions, protein–nucleic acid interactions, and posttranslational modifications, may also function in photobodies. (Top left) Phytochrome photobodies may contain nucleic acids, as demonstrated by the co-localization of RNA with TZP-phyB nuclear bodies. (Top right) Phytochromes undergo posttranslational modifications such as SUMOylation, phosphorylation, and polyubiquination. Phytochromes target PIFs for phosphorylation and polyubiquitination. (Bottom left) Multivalent molecules, such as intrinsically disordered proteins (curved lines), may promote photobody formation.

Proteins that promote phase separation are typically multivalent and frequently contain intrinsically disordered regions (IDRs) or prion-like domains (Figure 4; Banani et al., 2017). Multivalent molecules can undergo inter- or intra- molecular interactions, binding to multiple partners simultaneously (Harmon et al., 2017). It is important to note that multivalent interactions drive phase separation, not necessarily intrinsically disordered proteins (IDPs) (Martin and Holehouse, 2020). Phytochrome’s N-terminal extension (NTE) contains a predicted IDR, which may promote their condensation into photobodies (Burgie et al., 2021). Several proteins that colocalize to phyB photobodies are predicted to contain prion-like domains or IDRs, such as ELF3 and HY5 (Covarrubias et al., 2017; Cuevas-Velazquez and Dinneny, 2018; Jung et al., 2020). Proteins with IDRs in plant cells seem to play a role in different mechanisms underlying responses to environmental stimuli (Covarrubias et al., 2017).

The presence and concentration of key protein components contribute to the phase separation of condensates. As the abundance of nuclear Pfr increases with increasing red light intensity in a dosage dependent manner, large photobodies form, thus red light may promote the phase separation of photobodies *in vivo* (Figure 3). Conversely, as warm temperatures lead to the thermal reversion of Pfr back to Pr and the dissipation of photobodies, temperature may also regulate the phase separation of photobodies *in vivo* (Figure 3). Models that have been used to describe biomolecular condensate formation are the “stickers and spacers” model and the “scaffold and client” model (Semenov and Rubinstein, 1998; Banani et al., 2017; Posey et al., 2018; Wang A. et al., 2018; Holehouse, 2019). In the former model, stickers are portions of an amino acid sequence that promote intermolecular interactions, while the spacers are regions that are inert in promoting interactions (Wang A. et al., 2018). In the later model, scaffold proteins are necessary for condensate formation to occur, whereas client proteins localize to the condensate but are not necessary or sufficient for formation (Banani et al., 2017). In the context of photobodies, it may be helpful to think of HMR, PCH1, RCB, or NPC as ‘scaffold’ proteins, as they are essential for proper photobody formation (Figure 3). In contrast, the proteins that are shown to colocalize but are not necessary for formation may be thought of as ‘clients’. Other factors, such as pH, temperature, salinity, and environmental factors, also contribute to phase separation and formation of condensates (Yoo H. et al., 2019).

In addition, posttranslational modifications, such as SUMOylation, phosphorylation, and ubiquitination, are found to be associated with condensate formation or dissolution (reviewed in Owen and Shewmaker, 2019). Phytochromes are SUMOylated, phosphorylated, and ubiquitinated (Quail et al., 1978; Kim et al., 2004; Jang et al., 2010; Sadanandom et al., 2015; Figure 4). SUMOylation is shown to modulate far-red light-induced phyA signaling (Qu et al., 2020). PhyB is SUMOylated at its C-terminal module, which is enhanced by red light (Sadanandom et al., 2015). Since phyB’s C-terminal module is required for photobody formation, there may be a potential connection between this posttranslational modification and photobodies. (For a review on plant SUMOylation, readers are pointed to reviews by Augustine and Vierstra, 2018; Srivastava et al., 2021). Phosphorylation of phytochromes at serine residues attenuates light signaling and interrupts protein-protein interactions with downstream partners (reviewed in Hoang et al., 2019). For example, phosphorylation of phyB^Ser86^ accelerates its thermal reversion and inhibits binding with PIF3 (Medzihradszky et al., 2013). The reversion of Pfr back to Pr in thermal reversion leads to the disassembly of photobodies; therefore, phyB’s phosphorylation may be connected to photobody disassembly. Furthermore, nuclear phytochrome is targeted for polyubiquitination by COP1 in a red light and PIF mediated manner (Jang et al., 2010). Like other biomolecular condensates, the posttranslational modifications of phytochromes or binding partners could potentially be associated with photobody formation or dissipation (Figure 4).

Biomolecular condensates undergoing LLPS is an emerging topic in the plant field, and there are a growing number of studies on plant biomolecular condensates demonstrating LLPS (Fang et al., 2019; Powers et al., 2019; Dorone et al., 2020; Jung et al., 2020; Ouyang et al., 2020; Zavaliev et al., 2020; Huang et al., 2021). One of these recent findings was on a phyB interacting protein, ELF3, demonstrating that ELF3 nuclear bodies form via LLPS (Jung et al., 2020). Through biochemical and phenotypic analysis, Jung et al. (2020) demonstrated that the *Arabidopsis* ELF3 prion-like domain is necessary and sufficient for its temperature responsive phase separating ability *in vivo* and *in vitro*. ELF3’s prion-like domain was shown to regulate thermoresponsive binding of ELF3 to target genes and flowering time (Jung et al., 2020). LLPS may be the mode by which ELF3 connects temperature signals with the circadian clock (Jung et al., 2020; Wilkinson and Strader, 2020). The field is just beginning to understand how plant biomolecular condensates form. Overall, there is a whole realm of exciting questions awaiting to be answered regarding the formation process of plant biomolecular condensates.

## Photobody Functions

The functions of nuclear condensates include, but are not limited to, protein/nucleic acid sequestration, protein ubiquitination, transcription regulation, RNA processing, and organizing genome structure (Sabari et al., 2020). To date, the function of photobodies has yet to be clearly defined. However, their hypothesized functions overlap with functions associated with other nuclear condensates, including protein storage, proteolysis, protein sequestration, and gene regulation (Van Buskirk et al., 2012).

The first hypothesized function is that nuclear bodies act as storage sites to stabilize Pfr and prevent phyB from reverting back to Pr (Rausenberger et al., 2010; Van Buskirk et al., 2014). In agreement with this, PCH1, one of the essential components of photobodies, stabilizes phyB-Pfr, slowing its thermal reversion rate and stabilizing phyB photobodies in the dark (Huang et al., 2016, 2019; Enderle et al., 2017). It is likely that being a storage center for active phyB is not the only role that photobodies play.

There is a strong line of evidence for the second hypothesized function that photobodies are sites for protein degradation. HMR is an essential component of photobodies and is critical for phyA, PIF1, and PIF3 degradation (Chen et al., 2010; Galvão et al., 2012). Light induces phyA and phyB’s negative regulation of PIFs through phosphorylation, polyubiquitination, and 26S proteasomal degradation, possibly within nuclear bodies (Al-Sady et al., 2006; Lorrain et al., 2008; Dong et al., 2017). Conversely, PIF3 promotes the degradation of phyB (Ni et al., 2013). Light-Response-Bric-a-Brack/Tamtrack/Broad (LRB1/2/3) promotes the polyubiquitination of phyB and PIF3 in a light-dependent manner (Christians et al., 2012; Ni et al., 2014). PIF3 enhances LRB2 binding to phyB-Pfr, which is required for proteasome-mediated phyB protein degradation (Christians et al., 2012). This negative regulatory mechanism was termed the mutually assured destruction model. In this model, phyB stimulates the phosphorylation of PIF3, promoting the binding to LRBs, which leads to the polyubiquitination of both phyB and PIF3, targeting them for 26S proteasomal degradation in a light-dependent manner (Ni et al., 2014). This bidirectional feedback loop of PIF3 and phyB proteolysis may occur within photobodies since their co-localization into early bodies is associated with PIF3 degradation (Bauer et al., 2004). Similarly, blue light-induced CRY2 nuclear bodies are involved in degradation via its association with the 26S proteasome (Shalitin et al., 2003; Yu et al., 2007, Yu et al., 2010; Wang et al., 2015; Liu et al., 2017). Overall, these light-mediated nuclear bodies may be sites for proteasomal degradation in light signaling.

A third hypothesized function of photobodies is to sequester proteins to modulate signaling. PhyA and phyB colocalize with SPA1 into nuclear bodies, which are important for the phyA-SPA1 interaction (Lu et al., 2015). Light activation of phyA nuclear bodies prevents COP1-SPA1-mediated degradation of LONG HYPOCOTYL IN FAR-RED 1 (HFR1), a promoter of photomorphogenesis (Sheerin et al., 2015). Similar to phyA bodies and their ability to block COP1-SPA1 activity, CRY1 co-localizes and directly interacts with SPA1 in CRY-bodies, attenuating the COP1-SPA1 interaction and negatively regulating COP1, albeit in a blue light-dependent manner (Lian et al., 2011; Zuo et al., 2011).

Lastly, there is evidence that photobodies may be involved in gene regulation. PhyB has been shown to regulate transcription, alternative splicing, and alternative promoter selection to mediate light responses, which could take place within photobodies (Shikata et al., 2014; Ushijima et al., 2017; Dong et al., 2020). Splicing factors, transcription factors, and transcriptional regulators are shown to colocalize to phyB-photobodies, suggesting a potential overlap of transcriptional and post-transcriptional regulation within these subnuclear compartments (Van Buskirk et al., 2012; Xin et al., 2017). One compelling example of photobodies demonstrating transcriptional activity are TZP-phyB bodies. TZP-phyB nuclear bodies are shown to serve as sites of active transcription, activating gene expression to promote flowering (Kaiserli et al., 2015). TZP is an RNA binding protein, and TZP-phyB bodies are shown to associate with RNA (Kaiserli et al., 2015; Figure 4). There are many examples of biomolecular condensates associating with nucleic acids, particularly RNA, such as paraspeckles and P granules (reviewed in Roden and Gladfelter, 2021). Since many condensates containing RNA binding proteins are involved in RNA processing, TZP bodies may also be involved in RNA metabolism. Similarly, blue light-induced CRY nuclear bodies are associated with transcription regulation (Wang Q. et al., 2018).

As there is supporting evidence for all hypothesized functions, including sites for storage, proteolysis, protein sequestration, and transcription regulation, photobodies likely have multiple functions within the cell to shape plant responses to various environmental stimuli.

## Research Directions and Outstanding Questions

There has been a recent explosion of excitement and interest in biomolecular condensates across kingdoms. The idea that photobodies may be undergoing LLPS is not new (Cuevas-Velazquez and Dinneny, 2018). However, the idea that phase separation of biomolecular condensates connects cellular signaling in plants with the external environment is novel.

Phytochrome photobodies may serve as a controllable system to study LLPS *in vivo* and *in vitro* through its reversible formation and dissipation in response to environmental and cellular factors (Figure 3). Optogenetic tools use light to tightly control molecular and cellular signaling (Taslimi et al., 2014; Dine and Toettcher, 2018; Goglia and Toettcher, 2019). A recent optogenetic tool named OptoDropletTFs uses the *Arabidopsis* photoreceptors CRY2 or phyB, through their light-induced oligomerization, as a molecular switch to induce LLPS (Schneider et al., 2021). Through the red light-inducible phyB-PIF6 interaction, this study made a phytochrome-based OptoDropletTF system by creating a phyB-IDR fusion protein to significantly increase transcriptional output via LLPS (Schneider et al., 2021). IDR-mediated phase separation may be a common mechanism in regulating transcription (Boija et al., 2018). As phyA, phyB, and PIFs are recruited to promoters to regulate transcription, LLPS may be occurring via the formation of photobodies to enhance these gene regulatory processes (Chen et al., 2010; Guo et al., 2012; Pfeiffer et al., 2012; Jung et al., 2016; Brodsky et al., 2020). Overall, photobodies may provide a useful system in studying LLPS to regulate cellular processes.

Photobody function, composition, and biogenesis have yet to be clearly defined since their discovery in 1999. Photobodies may be central points for regulating, organizing, and tightly coordinating the complex interception of phytochrome-mediated processes: photomorphogenesis, de-etiolation, flowering, etc. There is likely not just a single type of ‘photobody’, but rather numerous nuclear bodies that organize the complex overlapping of light, temperature, and circadian signaling pathways. Investigating the molecular, cellular, and biophysical properties that lead to the formation of these nuclear condensates will provide great insight into a potentially conserved mechanism by which nuclear condensates form. A combination of high-resolution microscopy, proteomic, genomic, structural, computational, and biophysical approaches will begin to answer questions such as: How do phytochrome photobodies form? What is their function? What is their composition? And can they be manipulated to improve crop fitness? It will be very exciting to see what new information is discovered in the coming years.

There are boundless avenues for future research to further explore and characterize photobody biomolecular condensates through the lens of LLPS. Below are questions and topics that will be of interest:

• How can our understanding of photobodies and their role as environmental sensors be used to improve crops through altering shade, temperature, and daylength sensitivity?
• Are there multiple different types of photobodies present simultaneously in a single nucleus?
• Is there a physical interaction between nucleolar associated photobodies and the nucleolus?
• Do nucleolar associated photobodies share commonalities with Cajal bodies, which frequently associate with the nucleolus (Monneron and Bernhard, 1969; Trinkle-Mulcahy and Sleeman, 2017)?
• Does LLPS occur *in vivo* during phyB and PIF transcription regulation?
• Are there other nuclear bodies that associate with nucleic acids? If so, are target genomic loci recruited to photobodies for transcription?
• Are posttranslational modifications associated with photobody formation or dissipation?
• Can the effects of photobodies seen so far be recapitulated with phyB expression at endogenous levels?

## Author Contributions

SP and DN wrote the manuscript. Both authors contributed to the article and approved the submitted version.

## Conflict of Interest

The authors declare that the research was conducted in the absence of any commercial or financial relationships that could be construed as a potential conflict of interest. The handling editor declared a past co-authorship with one of the author DN.

## Publisher’s Note

All claims expressed in this article are solely those of the authors and do not necessarily represent those of their affiliated organizations, or those of the publisher, the editors and the reviewers. Any product that may be evaluated in this article, or claim that may be made by its manufacturer, is not guaranteed or endorsed by the publisher.
